# Supplementary material for: Bacteriophage therapy for the treatment of Mycobacterium tuberculosis infections in humanized mice
Source: Commun Biol. 2024 Mar 9;7:294. doi: 10.1038/s42003-024-06006-x (PMC10924958; doi:10.1038/s42003-024-06006-x)
Supplement: Supplementary file 2 — Supplementary Figs. [file 42003_2024_6006_MOESM2_ESM.pdf]

Supplementary Figures

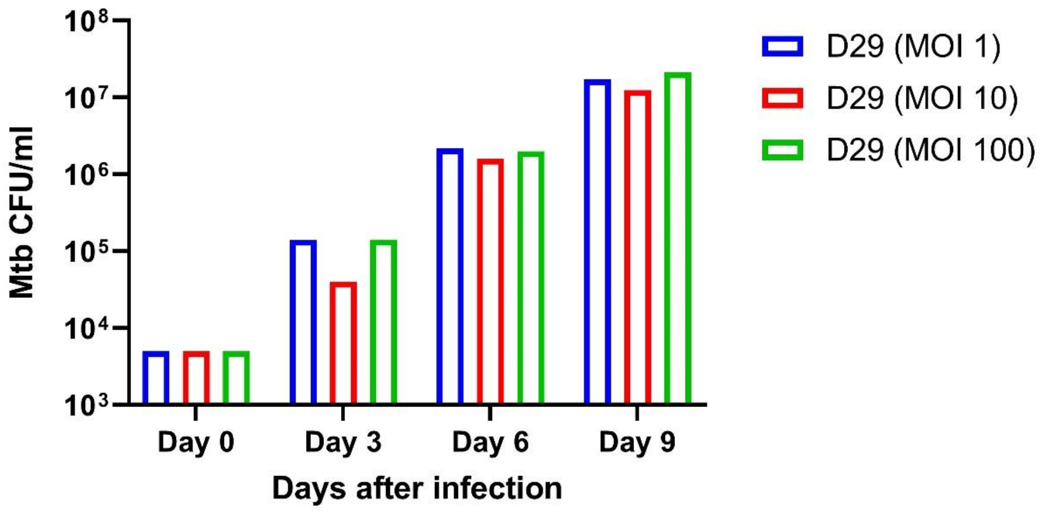

Supplementary Figure 1: Testing of the capacity of the D29 phage to eliminate *Mtb* at different MOIs in liquid culture. H37Rv (1 x 10^5 CFU) were inoculated with D29 phage at an MOI of 1, 10 and 100, respectively. Afterwards, the mix was inoculated in 20 mL of 7H9 culture media and incubated at 37°C. One mL of bacterial culture was recovered every 3 days and 10-fold serial dilutions were plated in 7H10 agar plates for CFU counting after 2 weeks of incubation.

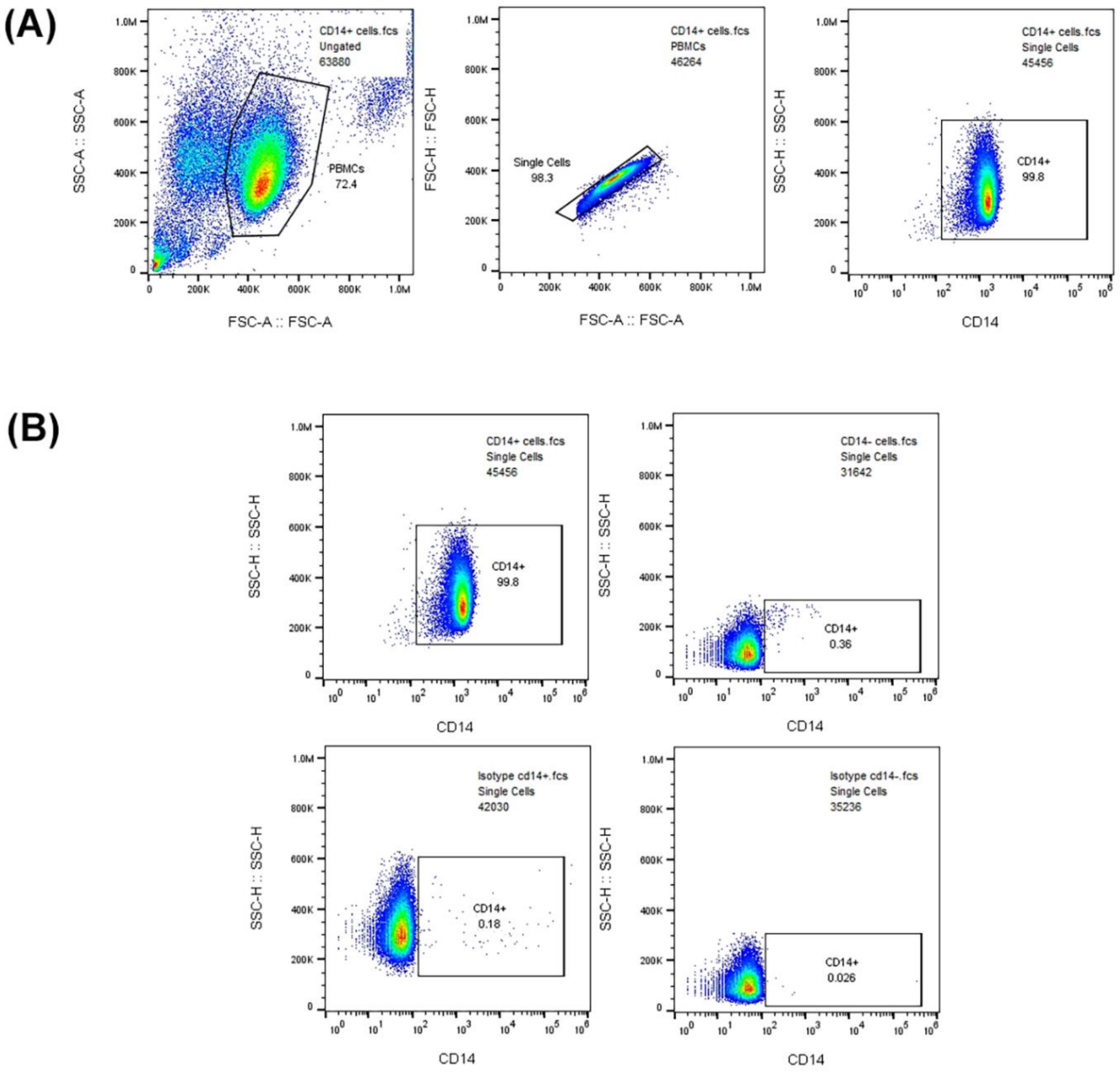

Supplementary Figure 2: The purity of microbeads isolated CD14+ cells. (A) Gating strategy. (B) The purity of CD14+ cells. The top panel shows the percentages of CD14+ cells (left) and CD14- cells (right). The bottom panel shows the isotype controls of CD14+ and CD14- cells.

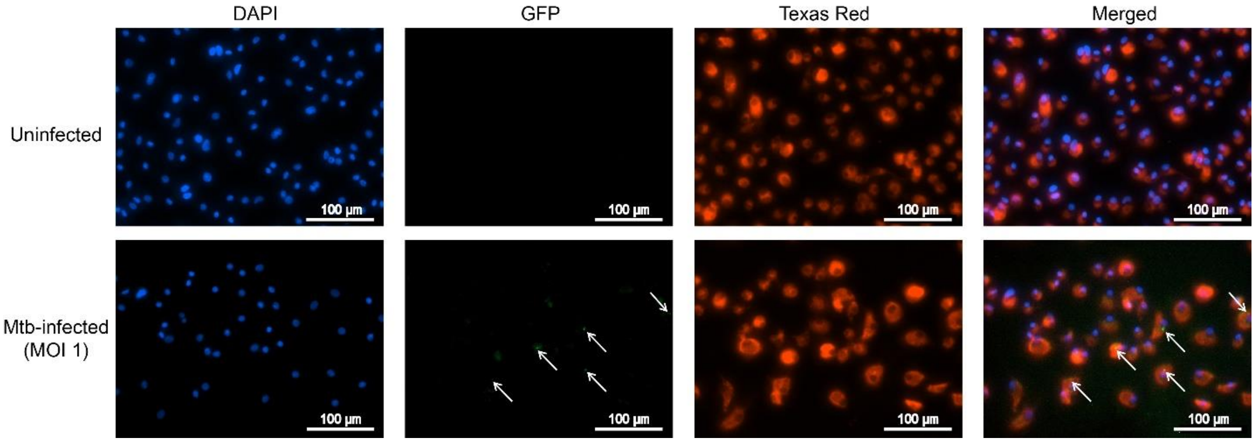

Supplementary Figure 3: Intracellular infection of human macrophages with GFP-expressing *Mtb*. Primary human macrophages were infected with H37Rv-GFP at an MOI of 1. Four hours later, the media was removed, and the cells were rigorously washed for 3 times with PBS to remove extracellular bacteria, followed by incubation for 10 min at 37 °C in 1 ml of CellMask™ Deep red Plasma Membrane Stain dilution. The cells were then fixed with 4% paraformaldehyde for 30 minutes at room temperature and permeabilized for 10 min using 0.1% (vol/vol) Triton X-100 diluted in PBS. The macrophages were blocked by incubation with 2% BSA in PBS at room temperature for 1 hour. The slides were mounted using DAPI-supplemented mounting medium (Abcam, Cambridge, UK) and images were captured with a LionheartLX automated microscope (Biotek, Winoovski, VT). Fluorescent images in the blue (DAPI), green (GFP) and Texas red (Cellmask™) channels were acquired for uninfected (upper panel) and Mtb-infected (lower panel) macrophages, and processed with the GEN5 software version 3.09 (Biotek). The white arrows show the intracellular *Mtb*.

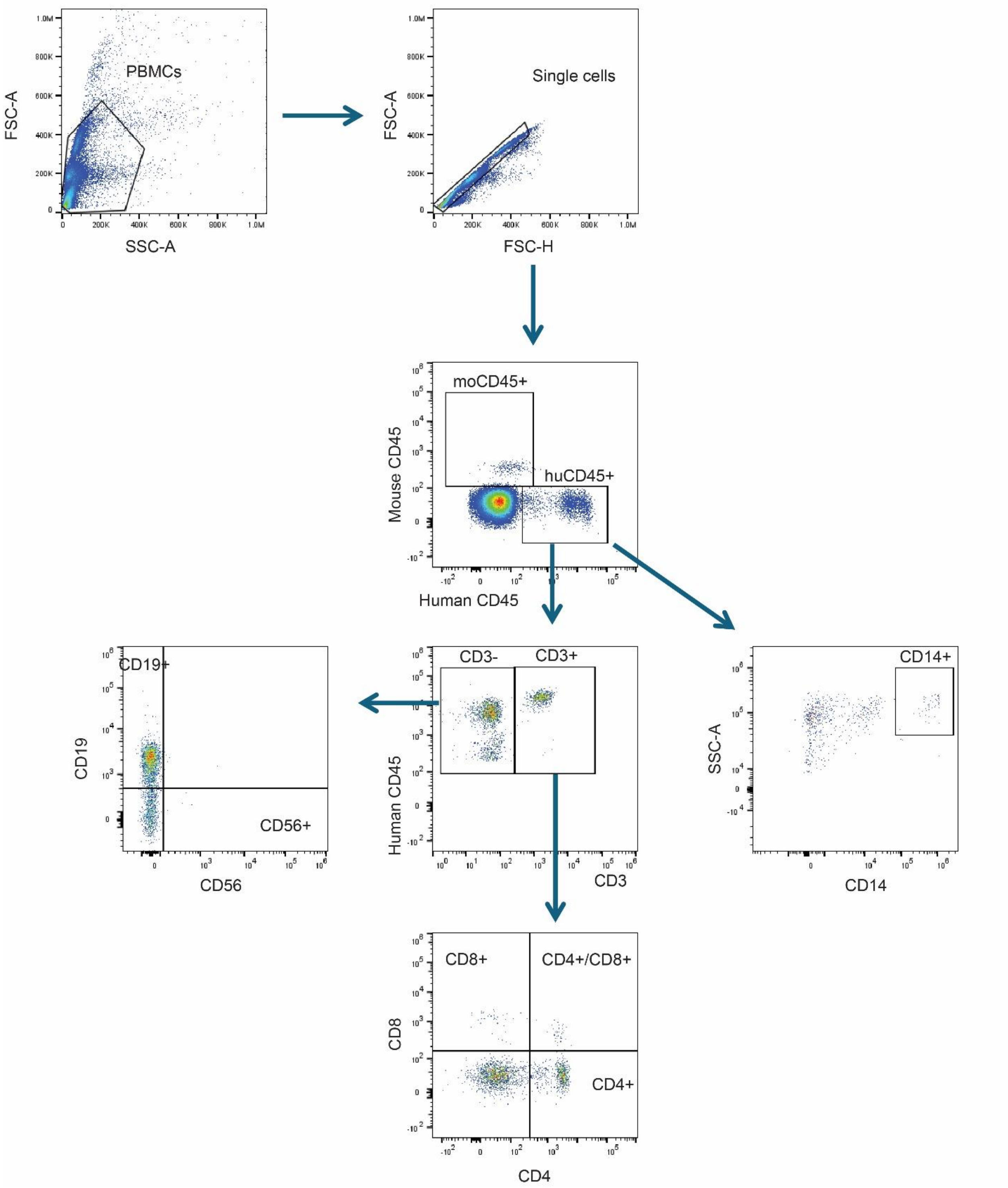

Supplementary Figure 4: Gating strategy for characterizing the differentiation of human immune cells in humanized NSG-SGM3 mice.

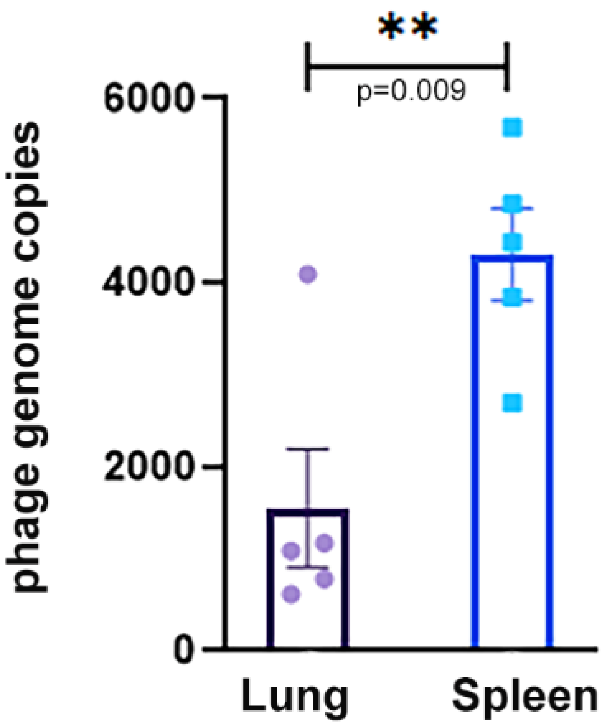

Supplementary Figure 5: Phage genome copy numbers in the lung and spleen homogenates of the phage-treated humanized mice. Five phage-treated mouse lung and spleen homogenates were used to isolate phage genomic DNA, and quantitative PCR was performed to determine the phage genome copy numbers in the tissue homogenates. Each dot represents the mean of triplicates, and paired T test was used to analyze the differences between the lung and spleen homogenates. Statistical significance was defined as *P≤0.05, **P≤0.01, and ***P≤0.001.

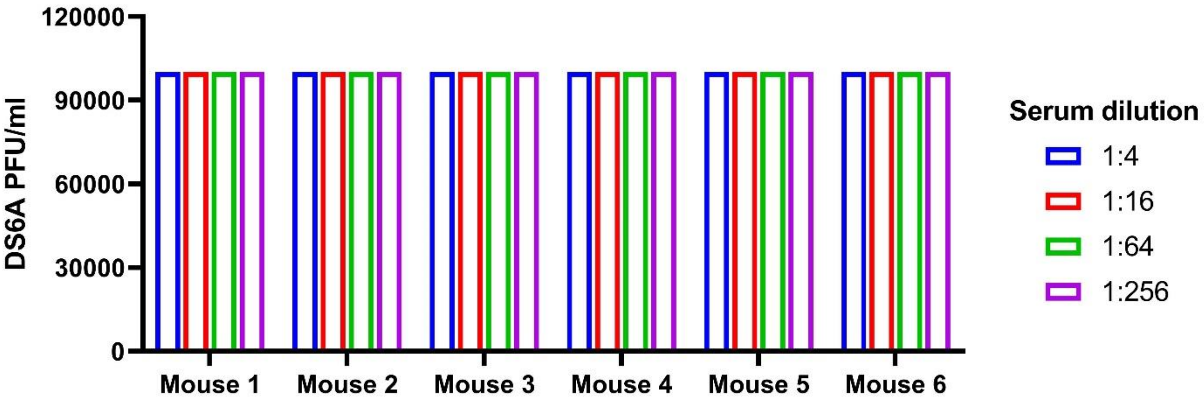

Supplementary Figure 6: The sera from phage DS6A-treated humanized mice did not show neutralizing capacity against phage DS6A. Serial dilutions of phage DS6A-treated humanized mouse sera were mixed with 2.5×10^4 phage DS6A and incubated for 1 h, then 200 µL of freshly prepared log-phase growth *Mtb* (OD value of 0.5) single cell suspension were added to the mixture and incubated at 37°C for an additional 10 min. The mixture was then added to 1.5 mL of melted top agar and poured in a six-well plate that was previously loaded with 7H10 agar to develop the phage plaques.

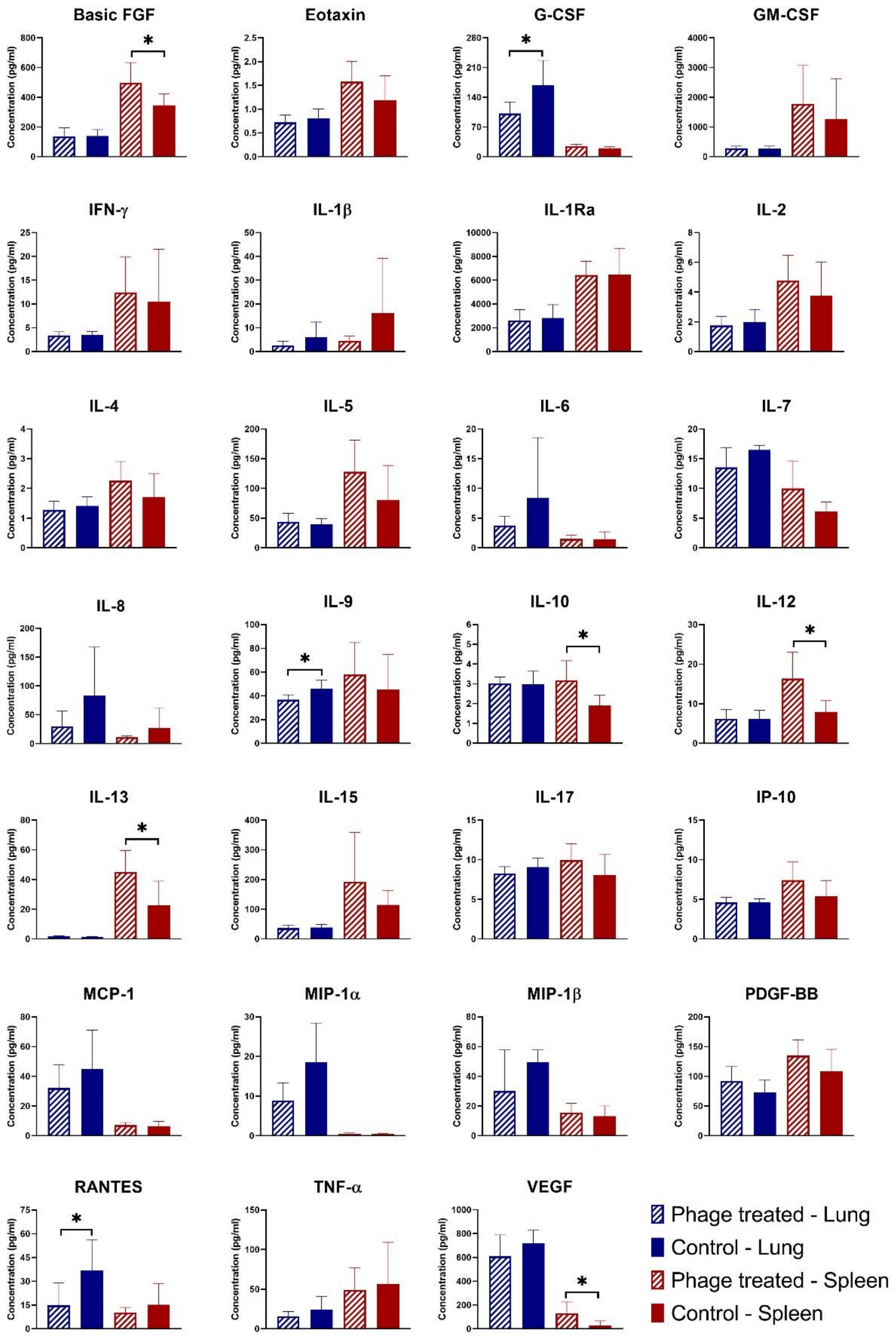

Supplementary figure 7: Cytokine profiles of the experimental mouse spleen and lung homogenates. Twenty-seven cytokines and chemokines of human lymphocytes and

myeloid cells in the lung and spleen homogenates of the humanized mice were determined by multiplex assay. Five mice from control group (*Mtb*-infected) and phage treated group were used for cytokine profiling. Unpaired T test was used to analyze the differences between control and phage-treated group. Statistical significance was defined as *$P \leq 0.05$, **$P \leq 0.01$, and ***$P \leq 0.001$.
