# Supplementary material for: Bacteriophage therapy for the treatment of Mycobacterium tuberculosis infections in humanized mice
Source: Commun Biol. 2024 Mar 9;7:294. doi: 10.1038/s42003-024-06006-x (PMC10924958; doi:10.1038/s42003-024-06006-x)
Supplement: Supplementary file 5 — Reporting Summary [file 42003_2024_6006_MOESM5_ESM.pdf]

nature portfolio

Corresponding author(s): Guohua Yi, John J. Dennehy, Buka Samten

Last updated by author(s): Feb 20, 2024

Reporting Summary

Nature Portfolio wishes to improve the reproducibility of the work that we publish. This form provides structure for consistency and transparency in reporting. For further information on Nature Portfolio policies, see our Editorial Policies and the Editorial Policy Checklist.

Statistics

For all statistical analyses, confirm that the following items are present in the figure legend, table legend, main text, or Methods section.

n/a	Confirmed	
☐	☒	The exact sample size (*n*) for each experimental group/condition, given as a discrete number and unit of measurement
☐	☒	A statement on whether measurements were taken from distinct samples or whether the same sample was measured repeatedly
☐	☒	The statistical test(s) used AND whether they are one- or two-sided<br>*Only common tests should be described solely by name; describe more complex techniques in the Methods section.*
☒	☐	A description of all covariates tested
☒	☐	A description of any assumptions or corrections, such as tests of normality and adjustment for multiple comparisons
☐	☒	A full description of the statistical parameters including central tendency (e.g. means) or other basic estimates (e.g. regression coefficient) AND variation (e.g. standard deviation) or associated estimates of uncertainty (e.g. confidence intervals)
☐	☒	For null hypothesis testing, the test statistic (e.g. *F*, *t*, *r*) with confidence intervals, effect sizes, degrees of freedom and *P* value noted<br>*Give P values as exact values whenever suitable.*
☒	☐	For Bayesian analysis, information on the choice of priors and Markov chain Monte Carlo settings
☒	☐	For hierarchical and complex designs, identification of the appropriate level for tests and full reporting of outcomes
☐	☒	Estimates of effect sizes (e.g. Cohen's *d*, Pearson's *r*), indicating how they were calculated

Our web collection on statistics for biologists contains articles on many of the points above.

Software and code

Policy information about availability of computer code

Data collection	We searched GenBank for phage genomes.
Data analysis	GraphPad Prism v8

For manuscripts utilizing custom algorithms or software that are central to the research but not yet described in published literature, software must be made available to editors and reviewers. We strongly encourage code deposition in a community repository (e.g. GitHub). See the Nature Portfolio guidelines for submitting code & software for further information.

Data

Policy information about availability of data

All manuscripts must include a data availability statement. This statement should provide the following information, where applicable:

- Accession codes, unique identifiers, or web links for publicly available datasets
- A description of any restrictions on data availability
- For clinical datasets or third party data, please ensure that the statement adheres to our policy

All data supporting the findings of this study are available in the manuscript. If there are any special requests or questions for the data, please contact the corresponding author (G.Y.).

Human research participants

Policy information about studies involving human research participants and Sex and Gender in Research.

Reporting on sex and gender	Wherever possible, each animal experiment contains equal numbers of male and female mice to exclude sex bias.
Population characteristics	Healthy donors who are tuberculin-negative, don't have a history on HIV infection, anemia, liver or kidney diseases, and are not pregnant.
Recruitment	The employees and students within the University of Texas Health Science Center at Tyler
Ethics oversight	The Institutional Review Board (IRB) ethics committee of the UTHSCT

Note that full information on the approval of the study protocol must also be provided in the manuscript.

Field-specific reporting

Please select the one below that is the best fit for your research. If you are not sure, read the appropriate sections before making your selection.

☒ Life sciences ☐ Behavioural & social sciences ☐ Ecological, evolutionary & environmental sciences

For a reference copy of the document with all sections, see nature.com/documents/nr-reporting-summary-flat.pdf

Life sciences study design

All studies must disclose on these points even when the disclosure is negative.

Sample size	Power analyses were performed to determine the effect sizes.
Data exclusions	GraphPad Outlier Calculator was used to determine if the data need to be excluded. However, no data was excluded in the experiments.
Replication	All experiments were repeated at least once to ensure reproducibility.
Randomization	Animal experiments were designed in a randomized manner.
Blinding	Animal experiments were designed in a blind manner for groups.

Reporting for specific materials, systems and methods

We require information from authors about some types of materials, experimental systems and methods used in many studies. Here, indicate whether each material, system or method listed is relevant to your study. If you are not sure if a list item applies to your research, read the appropriate section before selecting a response.

Materials & experimental systems

n/a	Involved in the study	
☐	☒	Antibodies
☒	☐	Eukaryotic cell lines
☒	☐	Palaeontology and archaeology
☐	☒	Animals and other organisms
☒	☐	Clinical data
☒	☐	Dual use research of concern

Methods

n/a	Involved in the study	
☒	☐	ChIP-seq
☐	☒	Flow cytometry
☒	☐	MRI-based neuroimaging

Antibodies

Antibodies used	anti-human CD11b-APC (cat# 301350), anti-human CD14-FITC (cat# 325604), anti-human CD16-BV711 (cat# 302044), anti-human-CD45-BV421 (cat# 368521), anti-human CD3-PE (cat# 980008), anti-human CD4-Alexa Fluor 488 (cat# 300519), anti-human CD8-BV605 (cat# 344741), anti-human CD56-PE/Cy5 (cat#304607), anti-mouse IgG1-APC (cat# 400142), anti-mouse IgG1-FITC(cat# 400108), anti-mouse IgG1 BV711 (cat# 400168), anti-mouse IgG1 BV421 (cat# 406616), anti-mouse IgG1 PE (cat# 406607), anti-mouse IgG1 Alexa-fluor 488 (cat# 406626). All of these were purchased from BioLegend Inc.<br>anti-human CD19-AmCyan (cat# 339190), anti-mouse IgG1-BV605 (cat# 742477), anti-mouse IgG1-PE/Cy5 (cat# 550083), and anti-mouse IgG1-AmCyan (cat# 329196) were purchased from BD Biosciences.

goat anti-human IgG Fc-HRP (cat# ab98624), anti-human IgA alpha chain-HRP (cat# ab98558), and anti-human IgM mu chain-HRP (cat# ab98549) were purchased from Abcam Inc.

Validation

All purchased antibodies were validated in the vendor company with certificates. We further validated in the lab by staining and flow cytometry analyses.

Animals and other research organisms

Policy information about studies involving animals; ARRIVE guidelines recommended for reporting animal research, and Sex and Gender in Research

Laboratory animals

NSG-SGM3 mice (Strain #:013062, RRID:IMSR_JAX:013062) were purchased from Jachson Laboratory and bred in UTHSCT animal facility. 4-5 weeks of mice were used for humanization.

Wild animals

n/a

Reporting on sex

Wherever possible, each animal experiment contains equal numbers of male and female mice to exclude sex bias.

Field-collected samples

n/a

Ethics oversight

IACUC committee of the University of Texas Health Science Center at Tyler

Note that full information on the approval of the study protocol must also be provided in the manuscript.

Flow Cytometry

Plots

Confirm that:

- [x] The axis labels state the marker and fluorochrome used (e.g. CD4-FITC).
- [x] The axis scales are clearly visible. Include numbers along axes only for bottom left plot of group (a 'group' is an analysis of identical markers).
- [x] All plots are contour plots with outliers or pseudocolor plots.
- [x] A numerical value for number of cells or percentage (with statistics) is provided.

Methodology

Sample preparation

1. for human blood, the blood was used to isolate PBMCs, then the CD14+ cells were further isolated from these PBMCs using microbeads.
2. for humanized mouse blood, the blood was used to isolate PBMCs, and the PBMCs were used for immune cell analysis by flow cytometry.

Instrument

Attune Flow Cytometer

Software

Attune NxT, FlowJo v11.

Cell population abundance

The purity of CD14+ cells was >95%.

Gating strategy

gating on the human lymphocytes.

- [x] Tick this box to confirm that a figure exemplifying the gating strategy is provided in the Supplementary Information.
